# Hardware Sophistications in Subthalamic Nucleus Deep Brain Stimulation for Parkinson's Disease; Is the Juice Worth the Squeeze?

**DOI:** 10.1002/mdc3.70725

**Published:** 2026-07-02

**Authors:** Marwan Hariz, Patric Blomstedt

**Affiliations:** ^1^ Department of Clinical Science, Neuroscience Umeå University Umeå Sweden; ^2^ UCL Institute of Neurology London UK

**Keywords:** current steering, deep brain stimulation (DBS), directional electrodes, motor outcome, Parkinson's disease, subthalamic nucleus

## Abstract

**Background:**

New sophisticated deep brain stimulation (DBS) systems enabling steering of current and aiming at improving further motor outcome and decreasing side effects have virtually replaced old omnidirectional systems, despite being more labor‐intensive and expensive.

**Objectives:**

The objective was to evaluate whether modern directional DBS resulted in better motor improvement compared to “old” omnidirectional DBS.

**Methods:**

Publications on subthalamic nucleus (STN)‐DBS from the “old” period were compared to modern publications, focusing on severity of motor symptoms at baseline and motor outcome at various intervals after surgery.

**Results:**

Although patients from old DBS era had a more advanced disease at baseline than those treated with modern DBS hardware, they benefitted more from STN‐DBS and showed a higher percentage decrease in dopaminergic medication.

**Conclusions:**

The new directional DBS does not result in better motor outcome compared to the older omnidirectional DBS. However, it may, in some cases, contribute to decreasing some side effects of stimulation such as dysarthria.

The first report on subthalamic nucleus deep brain stimulation (STN‐DBS) for advanced Parkinson's disease (PD) was published in 1993.^1^ In March 1994, at the 11th Parkinson's Disease symposium in Rome, Grenoble neurologist Pierre Pollak presented the results of STN‐DBS, including videos of the first operated patients. The late professor David Marsden, who was present in the audience, publicly stated that this was “the most important discovery since levodopa.”^2^

In a meta‐analysis of outcomes of STN‐DBS published in 2006, totaling 961 patients with a mixed follow‐up of 6–60 months, the mean percentage improvement in off‐medication (off‐med) on‐stimulation (on‐stim) motor scores of the Unified Parkinson's Disease Rating Scale Part III (UPDRS‐III) was 52.3%, and the decrease in levodopa‐equivalent daily doses (LEDD) was 55.9%.^3^ Subpopulations of the same patients were followed at various interval, up to 15 years postsurgery, showing a decrease in efficacy but still decent motor outcome and relatively stable decrease in LEDD.^4^

These early patients who had STN‐DBS were in a rather advanced stage of the disease with a preoperative off‐med UPDRS‐III score around 50. The DBS hardware available at that time consisted of quadripolar leads and single‐ or dual‐implantable pulse generators (IPGs) that were programmed using a so‐called monopolar review to establish the optimal electrode contact and titrate the voltage until adequate clinical response was achieved.

The success of DBS encouraged industry to market new DBS systems featuring increased sophistication, including directional electrode contacts enabling steering of current in lateral directions and IPGs allowing a wider range of stimulation parameters, all with the declared aim of further improving the motor outcomes and decreasing side effects compared to older brands.^5^, ^6^ These sophistications prolonged time needed to properly program the stimulation.^7^, ^8^, ^9^ Starting in 2015, and notwithstanding their complexity and higher costs, directional DBS leads have progressively replaced the old conventional omnidirectional leads in patients with PD undergoing STN‐DBS.^7^, ^8^

The present contribution by two clinicians with three decades of clinical and academic experience in DBS discusses whether the technological sophistications and complexity of directional DBS translated into better motor improvement in patients compared to what was achieved using the “older” DBS systems.

## Patients and Methods

To investigate the potential added motor improvement enabled by newer DBS devices compared to older ones, the authors surveyed pertinent old and new publications on STN‐DBS, focusing on “the most common and consistent evaluation of motor function,”^10^ which is the change in off‐med UPDRS‐III scores and its corollary, the change in LEDD, between preoperative baseline and various lengths of follow‐up. We searched as much as possible for publications from various centers and various countries that reported not only the percentage improvement in UPDRS‐III motor scores but also the absolute motor scores at the preoperative baseline and at follow‐ups, to establish the severity of PD in patients operated with old‐generation nondirectional DBS versus those operated with newer directional DBS systems.

## Results

For the 6–12‐month follow‐up of old DBS, we analyzed 21 pioneering reports (803 patients) from 11 countries, published between 1998 and 2013, including one international multicenter study. The mean off‐med UPDRS‐III score was 51.23 at baseline and 24.11 at follow‐up (52.99% improvement). The mean decrease in LEDD was 53.75% (Supplemental File S1, Table S1).

For the 16 month–3 year follow‐up of old DBS, we analyzed 10 reports (482 patients) from six countries, published between 1999 and 2016, including two multicenter studies. The mean off‐med UPDRS‐III score was 47.16 at baseline and 28.38 at follow‐up (39.93% improvement). The mean decrease in LEDD was 50.6% (Supplemental File S1, Table S2).

For the 4–6‐year follow‐up of old DBS, we analyzed 11 reports (381 patients) from six countries, published between 2003 and 2015, including one international multicenter study. The mean off‐med UPDRS‐III score was 50.7 at baseline and 26.4 at follow‐up (46.9% improvement). The mean decrease in LEDD was 47.52% (Supplemental File S1, Table S3).

For the 8–11‐year follow‐up of old DBS, we considered seven reports (122 patients) from five countries, published between 2010 and January 2026, including one multicenter study. The mean off‐med UPDRS‐III score was 52.2 at baseline and 34.7 at follow‐up (33.1% improvement). The mean decrease in LEDD was 40.7% (Supplemental File S1, Table S4).

Notwithstanding the great attrition of patients over prolonged follow‐up, the baseline UPDRS‐III scores were constant, around 50, whereas at each follow‐up, the percentage improvement worsened as expected but was still a decent 33% at 8–11 years compared to baseline, and the percentage decrease in LEDD was roughly maintained. A summary of the evolution of these results over time is shown in Supplemental File S1, Table S5.

Concerning results of patients operated with new DBS systems, the publications providing the data needed for the present survey were as follows:

For the 3–12‐month follow‐up, we reviewed nine reports (918 patients) from three countries, published between 2012 and January 2026, including five multicenter studies. The mean off‐med UPDRS‐III score was 42.26 at baseline and 25.74 at follow‐up (38.43% improvement). The mean decrease in LEDD was 37.55% (Supplemental File S1, Table S6).

For the 5‐year follow‐up, the only available report was a US multicenter study.^11^ Table 1 shows the results from that study, as well a comparison of outcomes between old and new DBS hardware at the 3–12‐month and at 4–6‐year follow‐ups.

**TABLE 1:** Follow‐up at 3–12 months and at 4–6 years in patients with “old” deep brain stimulation (DBS) versus “new” DBS hardware

	F‐U 6–12 Month Old DBS	F‐U 3–12 Month New DBS	*P*‐Value Old vs. New DBS at 3–12 Month F‐U	F‐U 4–6 Year Old DBS Mean ± SD (Range)	F‐U 4–6 Year New DBS^a^
No patients	803	918		381	122
Mean off‐med (95% CI) baseline UPDRS‐III scores	50.91 (48.5–53.29)	42.09 (39.1–45.07)	*P* < 0.0001	50.7 ± 8.05 (36.4–68.3)	42.8 ± 9.4
Mean off‐med on‐stim (95% CI) UPDRS‐III scores	23.88 (21.63–26.13)	25.40 (22.50–28‐29)	*P* = 0.370	26.4 ± 4.74 (18–33.7)	27.6 ± 11.6
Mean ± SD% improvement in UPDRS‐III scores (range)	52.99 ± 11.89 (25–71)	38.43 ± 10.34 (18.2–63.3)	<0.001	46.9 ± 10.61 (29.6–63)	35.5
Mean ± SD% LEDD decrease (range)	53.75 ± 16.29 (19–84)	37.55 ± 19.32 (7.4–64)	*P* = 0.021	47.5 ± 11.06 (26–63)	28.3

*Note*: Mean values and 95% confidence interval (CI) of baseline off‐medication (off‐med) Unified Parkinson's Disease Rating Scale Part III (UPDRS‐III) scores and mean values and 95% CI of off‐med on‐stimulation (on‐stim) scores. Mean ± standard deviation (SD) (range) *percentage* change in UPDRS‐III scores and mean ± SD (range) *percentage* change in levodopa‐equivalent daily doses (LEDD). For statistical comparison between old DBS UPDRS‐III scores at baseline and new DBS UPDRS‐III scores at baseline, and between old DBS UPDRS‐III scores at 6–12 months versus old DBS UPDRS‐III scores at 3–12‐month follow‐up (F‐U), a subgroup meta‐analysis using random‐effects model was performed. For statistical comparison of *percentage* decrease in LEDD, the 2‐tailed Mann‐Whitney *U* test was used. Because for the 4–6‐year F‐U, only data from one study with new DBS hardware were available,^11^ no statistics could be used for comparison with the corresponding old DBS data at the 4–6‐year F‐U. The statistical pooling of percentage improvement in UPDRS‐III scores was strictly performed on the raw continuous data, so these percentages represent a postanalysis conversion of the weighted estimates. As for the pooled estimate of the percentage LEDD reduction, these are given as a purely descriptive synthesis.

^a^ Only one study using a new brand of DBS reported a 5‐year follow‐up.^11^

For the statistical evaluation of UPDRS III raw scores at baseline and follow‐up in patients treated with old DBS versus patients with new DBS, we performed a subgroup meta‐analysis using a random‐effects model (DerSimonian–Laird approach) to account for between‐study heterogeneity. (Details of the meta‐analysis, including forest plots, are available in Supplemental File S2).

## Discussion

The patients with old DBS had a more advanced disease than those with new DBS, judging by the difference in absolute motor scores off‐med at baseline. This reflects the more recent tendency to operate earlier in the disease process. Also, the percentage decrease in UPDRS‐III scores, as well as the percentage decrease in LEDD, was significantly more pronounced in the old DBS patients. On the contrary, the absolute UPDRS‐III scores off‐med on‐stim at 3–12‐month follow‐up were not different between the groups. It seems that in terms of UPDRS‐III improvement, STN‐DBS has reached a plateau regardless of which DBS system was used and whether patients were operated early or late in the disease process.

Systematic reviews and meta‐analyses, as well as reports from individual centers using directional DBS systems, have shown similar efficacy on decrease in UPDRS‐III scores off‐med when comparing directional stimulation to classical ring‐mode stimulation.^7^, ^12^, ^13^, ^14^ A recent systematic review concluded that directional DBS “is widely accepted, but clinical data justifying its increased complexity and cost are currently sparse.”^7^ Another paper reviewing meta‐analysis of outcome of old DBS, compared to a multicenter study using directional DBS, stated: “it is rather surprising that the recent very convincing results of modern surgery with its technological advances are still comparable to the efficacy and safety results of the pioneering team from Grenoble.”^15^

Although the new DBS systems enable directional steering of stimulation, this tool is not always used. Indeed, several surveys of programming practice of new DBS systems have indicated that the initial programming did not use directionality but still relied on the classical monopolar omnidirectional review, because using directional leads is complex and time‐consuming.^7^, ^8^, ^9^, ^16^, ^17^ Nonetheless, several studies have shown that the main incentive to prefer the directional DBS hardware is the possibility to correct for suboptimally placed electrodes or manage stimulation‐induced side effects, thereby enabling an expansion of the therapeutic window.^7^, ^8^, ^9^, ^14^, ^17^, ^18^, ^19^, ^20^

Observers who have been practically involved in STN‐DBS for decades, and who have been following the rich literature on this procedure since more than 30 years, cannot but make some astute reflections about these aspects:

First, it seems that with the advent of directional DBS, people are “discovering” a lot of various side effects of stimulation, especially issues with dysarthria and gait. Where were all these side effects during the height of STN‐DBS which was performed using the old DBS electrodes? The seminal multicenter study from 2001—the study on the basis of which STN‐DBS received FDA approval in the United States—listed only one single stimulation‐induced dysarthria in 102 patients with bilateral STN‐DBS!^21^ Another multicenter study published just a couple of years ago denied the occurrence of stimulation‐induced dysarthria in STN‐DBS patients stimulated with old‐brand DBS and followed for 2 years.^22^ Is it so that these side effects have been more or less overshadowed by the great motor benefits of STN‐DBS? Or was their occurrence simply downplayed then but resurfaced today to provide a rationale for the new DBS devices?

Be it as it may, there is no doubt that steering the current and directionality can be beneficial in some patients to alleviate some of the common side effects of DBS, such as dysarthria. In other cases, a change from omnidirectional to directional stimulation and using shorter pulse width can enhance the stimulation effect at longer follow‐up when an increase in previous omnidirectional stimulation is limited by side effects.^7^, ^8^, ^14^, ^17^, ^19^, ^20^

Ultimately, the benefit of modern DBS with current steering versus established omnidirectional stimulation must be decided through a randomized double‐blind multicenter trial comparing the two. A trial protocol published recently by the Amsterdam group aims to address explicitly whether “directional DBS for Parkinson's disease results in a better clinical outcome when compared to ring‐mode DBS.”^23^

Finally, the issue of adaptive closed‐loop DBS is still in their infancy and therefore not addressed in this brief report.

## Conclusion

Directional DBS leads have practically replaced omnidirectional leads in STN‐DBS despite not providing better motor improvement, either at short‐term or at longer‐term follow‐up. It seems that STN‐DBS as a treatment of PD had already reached a plateau of efficacy for motor symptoms with the old DBS systems. The exploration of eventual full benefit of directionality through current steering is laborious and time intensive, and perhaps the full potential of directional DBS is not always realized due to these constraints. So far, experience shows that these new tools can alleviate some stimulation side effects in some patients on chronic DBS, allowing an expansion of the therapeutic window. Whether these benefits will prove to be long lasting, and whether the juice is worth the squeeze, is still to be determined.

## Author Roles

(1) Research project: A. Conception, B. Organization, C. Execution; (2) Statistical analysis: A. Design, B. Execution, C. Review and critique; (3) Manuscript preparation: A. Writing of the first draft, B. Review and critique.

M.H.: 1A, 1B, 1C, 2A, 2B, 2C, 3A, 3B

P.B.: 1A, 1B, 2C, 3B

All authors take responsibility for the integrity of the data analysis.

## Disclosures

**Ethical Compliance Statement:** The present study is a viewpoint based on critical reading of the literature and does not include study or research on patients or animals and therefore is exempt from evaluation by an ethical committee. We confirm that we have read the journal's position on issues involved in ethical publication and affirm that this work is consistent with those guidelines.

**Funding Sources and Conflict of Interest:** None.

**Financial Disclosures for the Previous 12 Months:** M.H.: nothing to declare. P.B.: consultant for Abbott, Boston Scientific, Medtronic. Shareholder in Mithridaticum AB.

## Financial Disclosures and Conflicts of Interest

Author disclosures are available in the Supporting Information.

## Supporting information


**Supplemental File S1.** Supporting Information.
**Table S1.** “Old” deep brain stimulation (DBS); 6–12‐month follow‐up.
Twenty‐one publications on subthalamic nucleus‐DBS (STN‐DBS) between 1998 and 2013, using the “old” generation DBS hardware, with a 6–12 months of follow‐up (F‐U). Number of patients in each study; mean ± standard deviation (SD) Unified Parkinson's Disease Rating Scale Part III (UPDRS‐III) scores off‐med at baseline and mean ± SD UPDRS scores off‐med on‐stim at follow‐up; percentage motor improvement and percentage decrease in levodopa‐equivalent daily doses (LEDD). MC, multicenter.
**Table S2.** “Old” deep brain stimulation (DBS); 16 month–3 year follow‐up. Ten publications on subthalamic nucleus‐DBS (STN‐DBS) between 1999 and 2016, using the “old” generation DBS hardware, with a 16 month (mo)–3 years (yrs) follow‐up (F‐U). Number of patients in each study; mean Unified Parkinson's Disease Rating Scale Part III (UPDRS‐III) scores off‐med at baseline and mean UPDRS‐III scores off‐med on‐stim at follow‐up; percentage motor improvement and percentage decrease in levodopa‐equivalent daily doses (LEDD). Results from the “Earlystim” trial (in which STN‐DBS was performed on purpose earlier in the disease process) are included in this table. MC, multicenter.
**Table S3.** “Old” deep brain stimulation (DBS); 4–6‐year follow‐up. Eleven publications on subthalamic nucleus‐DBS (STN‐DBS) between 2003 and 2015, using the “old” generation DBS hardware, with a 4–6‐year (yrs) follow‐up (F‐U). Number of patients in each study; mean Unified Parkinson's Disease Rating Scale Part III (UPDRS‐III) scores off‐med at baseline and mean UPDRS‐III scores off‐med on‐stim at F‐U; percentage motor improvement and percentage decrease in levodopa‐equivalent daily doses (LEDD). MC, multicenter.
**Table S4.** “Old” deep brain stimulation (DBS); 8–11‐year follow‐up. Seven publications on subthalamic nucleus‐DBS (STN‐DBS) between 2010 and 2026, using the “old” generation DBS hardware, with 8–11 years (yrs) of follow‐up (F‐U). Number of patients in each study; mean Unified Parkinson's Disease Rating Scale Part III (UPDRS‐III) scores off‐med at baseline and mean UPDRS‐III scores off‐med on‐stim at F‐U; percentage motor improvement and percentage decrease in levodopa‐equivalent daily doses (LEDD).
**Table S5.** Summary of data from subthalamic nucleus deep brain stimulation (STN‐DBS) studies using the “old” DBS hardware. Mean Unified Parkinson's Disease Rating Scale Part III (UPDRS‐III) scores and percentage changes in UPDRS‐III and levodopa‐equivalent daily doses (LEDD) at various follow‐up intervals. The number of patients for each time period is the sum of patients of publication from Tables S1 to S4. Evidently, there is a great overlap of patients between publications from the same center, as the patients were followed at various follow‐up intervals, with an attrition in the number of patients over longer follow‐up periods.
**Table S6.** “New” deep brain stimulation (DBS); 3–12‐month follow‐up.
Nine publications on subthalamic nucleus‐DBS (STN‐DBS) between 2012 and January 2026, using “modern” DBS hardware, with a 3–12‐month follow‐up (F‐U). Number of patients in each study; mean ± standard deviation (SD) Unified Parkinson's Disease Rating Scale Part III (UPDRS‐III) scores off‐med at baseline and mean ± SD UPDRS‐III scores off‐med on‐stim at follow‐up; percentage motor improvement and percentage decrease in levodopa‐equivalent daily doses (LEDD).

**Supplemental File S2.** Meta‐analysis.
**Figure S1.** Preoperative baseline off‐med Unified Parkinson's Disease Rating Scale Part III (UPDRS III) scores in old deep brain stimulation (DBS) patients (blue) and preoperative baseline off‐med UPDRS III scores in new DBS patients (green).
**Figure S2.** Postoperative off‐med Unified Parkinson's Disease Rating Scale Part III (UPDRS III) scores in old deep brain stimulation (DBS) patients (blue) and postoperative off‐med on‐stim UPDRS III scores in new DBS patients (green).
**Table S1.** The meta‐analytic moderator analysis (old deep brain stimulation [DBS] vs. new DBS at baseline). Estimate is based on a random effect (RE) model using the DerSimonian–Laird approach. CI, confidence interval.
**Table S2.** The meta‐analytic moderator analysis (old deep brain stimulation [DBS] vs. new DBS at follow‐up). Estimate is based on a random effect (RE) model using the DerSimonian–Laird approach. CI, confidence interval.

**Data S3.** Coi_disclosure.

## Data Availability

The data that supports the findings of this study are available in the supplementary material of this article.
