# Supplementary material for: Hardware Sophistications in Subthalamic Nucleus Deep Brain Stimulation for Parkinson's Disease; Is the Juice Worth the Squeeze?
Source: Mov Disord Clin Pract. 2026 Jul 2:10.1002/mdc3.70725. Online ahead of print. doi: 10.1002/mdc3.70725 (PMC13337137; doi:10.1002/mdc3.70725)
Supplement: Supplementary file 1 — Supplemental File S1. Supporting Information. Table S1. “Old” deep brain stimulation (DBS); 6–12‐month follow‐up. Twenty‐one publications on subthalamic nucleus‐DBS (STN‐DBS) between 1998 and 2013, using the “old” generation DBS hardware, with a 6–12 months of follow‐up (F‐U). Number of patients in each study; mean ± standard deviation (SD) Unified Parkinson's Disease Rating Scale Part III (UPDRS‐III) scores off‐med at baseline and mean ± SD UPDRS scores off‐med on‐stim at follow‐up; percentage motor improvement and percentage decrease in levodopa‐equivalent daily doses (LEDD). MC, multicenter. Table S2. “Old” deep brain stimulation (DBS); 16 month–3 year follow‐up. Ten publications on subthalamic nucleus‐DBS (STN‐DBS) between 1999 and 2016, using the “old” generation DBS hardware, with a 16 month (mo)–3 years (yrs) follow‐up (F‐U). Number of patients in each study; mean Unified Parkinson's Disease Rating Scale Part III (UPDRS‐III) scores off‐med at baseline and mean UPDRS‐III scores off‐med on‐stim at follow‐up; percentage motor improvement and percentage decrease in levodopa‐equivalent daily doses (LEDD). Results from the “Earlystim” trial (in which STN‐DBS was performed on purpose earlier in the disease process) are included in this table. MC, multicenter. Table S3. “Old” deep brain stimulation (DBS); 4–6‐year follow‐up. Eleven publications on subthalamic nucleus‐DBS (STN‐DBS) between 2003 and 2015, using the “old” generation DBS hardware, with a 4–6‐year (yrs) follow‐up (F‐U). Number of patients in each study; mean Unified Parkinson's Disease Rating Scale Part III (UPDRS‐III) scores off‐med at baseline and mean UPDRS‐III scores off‐med on‐stim at F‐U; percentage motor improvement and percentage decrease in levodopa‐equivalent daily doses (LEDD). MC, multicenter. Table S4. “Old” deep brain stimulation (DBS); 8–11‐year follow‐up. Seven publications on subthalamic nucleus‐DBS (STN‐DBS) between 2010 and 2026, using the “old” generation DBS hardware, with 8–11 years [file MDC3-9999-0-s002.docx]

**SUPPLEMENTAL FILE 1: Tables 1-6 and their references**

**Supplemental Table 1: “old” DBS; 6-12 month follow-up**

Twenty-one publications on STN-DBS between 1998 and 2013, using the “old” generation DBS hardware, with a 6-12 months follow-up (F-U). Number of patients in each study; mean±SD UPDRS-III scores off-med at baseline and mean±SD UPDRS scores off-med on-stim at follow-up; percentage motor improvement and percentage decrease in levodopa equivalent daily doses (LEDD). MC: Multicenter

| **Author (site)** | **Year** | **No Pat** | **UPDRS-III Off-med pre-op** | **UPDRS-III Off-med On-stim** | **% change UPDRS-III scores** | **% decrease LEDD** | **F-U months** |
| --- | --- | --- | --- | --- | --- | --- | --- |
| Krack^1^ (Grenoble) | 1998 | 13 | 57.5 ±14.5 | 17 ±8.2 | 71 | 56 | 8 |
| Limousin^2^ (Grenoble) | 1998 | 20 | 59 ±10.1 | 30.1 ±16.2 | 49 | 49.75 | 12 |
| Kumar^3^ (Toronto) | 1998 | 7 | 55.7 ±12.1 | 19.4 ±6.7 | 65.2 | 40 | 6 |
| Moro^4^ (Rome) | 1999 | 7 | 67.9 ±10.0 | 39.8 ±12 | 41.3 | 64.5 | 12 |
| Pinter^5^ (Vienna) | 1999 | 9 | 49.4 ±14.1 | 27.1 ±7.1 | 55 | --- | 12 |
| Romito^6^ (Rome & Milan) | 2002 | 22 | 60.2 **± 12.3** | 29.9 ±10.9 | 50.2 | 69.3 | 12 |
| Anderson^7^ (Oregon) | 2005 | 10 | 51 ±13.0 | 27 ±10.9 | 48 | 38 | 12 |
| Houéto^8^ (Paris) | 2000 | 23 | 51.7 ±14.4 | 17 ±10.9 | 67 | 61 | 6 |
| Fraix^9^ (Grenoble) | 2000 | 24 | 54.4 ±13.1 | 18.2 ±8.8 | 68 | 76 | 12 |
| Molinuevo^10^ (Barcelona) | 2000 | 15 | 49.6 ±14.0 | 16.9 ±7 | 66 | 84 | 6 |
| Volkmann^11^ (Heidelberg/Köln) | 2001 | 16 | 56.4 ±11.62 | 22.4 ±16.93 | 60 | 65.3 | 12 |
| APD study group^12^ (MC) | 2001 | 91 | 54 ±15.0 | 25.7 ±14.1 | 51.3 | 37.3 | 6 |
| Ostergaard^14^ (Århus, Denmark) | 2002 | 26 | 51.3 ±12.1 | 18.3 ±10.0 | 64 | 19 | 12 |
| Simuni^15^ (Philadelphia) | 2002 | 12 | 43.5 ±3.6 | 23.9 ±2.2 | 47 | 55 | 12 |
| Bejjani^16^ (Paris) | 2000 | 12 | 50.7 ±4.3 | 18 **±**10.9 | 64 | 67 | 6 |
| Deuschl^17^ (Germany MC) | 2006 | 75 | 48 ±12.3 | 28.3 ±14.7 | 41 | 49 | 6 |
| Williams^18^ (UK MC) | 2010 | 178 | 47.6±14.0 | 30.6 ±15.2 | 33.6 | --- | 12 |
| De Gaspari^19^ (Milan) | 2006 | 12 | 33.5 ±12.9 | 15.7 ±7 | 53 | 62 | 12 |
| Odekerken^20^ (Netherlands MC) | 2013 | 63 | 44.4 ±15.5 | 24.1 ±14.4 | 45.7 | 43.5 | 12 |
| Follett^21^ (US) | 2010 | 147 | 43 ±$15.0$ | 32.2 ±16.2 | 25 | 31 | 6 |
| Wider^22^ (Swiss**)** | 2008 | 21 | 47.2 ±14.4 | 24.8 ±8.7 | 47.5 | --- | 6 |
| **Total no. of patients** |  | **803** |  |  |  |  |  |
| **Mean values ±SD**  **(range)** |  |  | **51.23±7.09**  **(33.5-67.9)** | **24.11±6.23**  **(15.7-39.8)** | **52.99±11.89**  **(25-71)** | **53.75±16.29**  **(19-84)** | **(6-12)** |

**Supplemental Table 2: “old” DBS; 16 month–3 years follow-up.**

Ten publications on STN-DBS between 1999 and 2016, using the “old” generation DBS hardware, with a 16 months (mo) to 3 years (yrs) follow-up (F-U). Number of patients in each study; mean UPDRS-III scores off-med at baseline and mean UPDRS-III scores off-med on-stim at follow-up; percentage motor improvement and percentage decrease in levodopa equivalent daily doses (LEDD). Results from the “Earlystim” trial (in which STN-DBS was performed on purpose earlier in the disease process), are included in this table.
MC = Multicenter.

| **Author (site)** | **Year** | **No Pat** | **UPDRS-III Off-med pre-op** | **UPDRS-III Off-med On-stim** | **% change UPDRS-III scores** | **% decrease LEDD** | **F-U mo / yrs** |
| --- | --- | --- | --- | --- | --- | --- | --- |
| Moro^4^ (Rome) | 1999 | 7 | 67 | 39.3 | 42 | --- | 16 mo |
| Romito^6^ (Rome, Milan) | 2002 | 7 | 60.8 | 31 | 49.1 | 69.6 | 3 yrs |
| Kleiner-Fisman^23^ (Toronto) | 2003 | 25 | 50.1 | 30.6 | 39 | 36 | 2 yrs |
| Vingerhoets^24^ (Lausanne) | 2002 | 20 | 48.8 | 26.9 | 45 | 79 | 21 mo |
| Pahwa^25^ (Kansas city) | 2003 | 19 | 41 | 29.5 | 28 | 57 | 28 mo |
| Weaver^26^ (US multicenter) | 2012 | 70 | 42.5 | 29.7 | 30 | 35 | 3 yrs |
| Follett^21^ (US multicenter) | 2010 | 147 | 43 | 32.1 | 25 | 31 | 2 yrs |
| Herzog^27^ (Kiel, Germany) | 2003 | 20 | 44.2 | 19.2 | 56.5 | 67 | 2 yrs |
| Odekerken^28^ (Netherlands MC) | 2016 | 43 | 41 | 28 | 31.7 | 42 | 3 yrs |
| Schüpbach^29^ (“Earlystim”) | 2013 | 124 | 33.2 | 17.5 | 53 | 39 | 2 yrs |
| **Total no. of patients** |  | **482** |  |  |  |  |  |
| **Mean values ±SD**  **(range)** |  |  | **47.16±10.05**  **(33.2-67)** | **28.38±6.26**  **(17.5-39.3)** | **39.93±11.01**  **(25-56.5)** | **50.6±17.77**  **(31-79)** | **(16mo-3yrs)** |

**Supplemental Table 3: “old” DBS; 4-6 years follow-up.**

Eleven publications on STN-DBS between 2003 and 2015, using the “old” generation DBS hardware, with a 4-6 years (yrs) follow-up
(F-U). Number of patients in each study; mean UPDRS-III scores off-med at baseline and mean UPDRS-III scores off-med on-stim at
F-U; percentage motor improvement and percentage decrease in levodopa equivalent daily doses (LEDD). MC = Multicenter.

| **Author (site)** | **Year** | **No Pat** | **UPDRS-III Off-med pre-op** | **UPDRS-III Off-med On-stim** | **% change UPDRS-III scores** | **% decrease LEDD** | **F-U years** |
| --- | --- | --- | --- | --- | --- | --- | --- |
| Rodriguez-Oroz^30^ (Pamplona) | 2004 | 10 | 49 | 18 | 63 | 50 | 4 |
| Rodriguez-Oroz^31^ (APD MC) | 2005 | 47 | 54.8 | 28.6 | 47.8 | 34.4 | 4 |
| Moro^32^ (APD MC) | 2010 | 35 | 57 | 28 | 49 | 34 | 5-6 |
| Krack^33^ (Grenoble) | 2003 | 49 | 55.7 | 25.8 | 54 | 63 | 5 |
| Aviles-Olmos^34^ (London) | 2014 | 41 | 50.3 | 33.7 | 33 | 45.1 | 5 |
| Gervais-Bernard^35^ (Lyon) | 2009 | 23 | 43.1 | 19.5 | 55 | 54.4 | 5 |
| Jiang (Guangdong^36^, China) | 2015 | 10 | 44.1 | 28.3 | 35.8 | 52.9 | 5 |
| Li^37^ (Beijing) | 2015 | 70 | 68.3 | 27.1 | 60.3 | 26 | 5 |
| Schüpbach^38^ (Paris) | 2005 | 30 | 51.9 | 26.2 | 49.5 | 58 | 5 |
| Kishore^39^ (Kerala, India) | 2010 | 45 | 36.4 | 22.1 | 39.3 | 48 | 5 |
| Wider^22^ (Lausanne) | 2008 | 21 | 47.2 | 33.2 | 29.6 | 57 | 5 |
| **Total no. of patients** |  | **381** |  |  |  |  |  |
| **Mean values ±SD**  **(range)** |  |  | **50.70±8.05**  **(36.4-68.3)** | **26.40±4.74**  **(18-33.7)** | **46.9±10.61**  **(29.6-63)** | **47.52±11.06**  **(26-63)** | **(4-6yrs)** |

**Supplemental Table 4: “old” DBS; 8-11 years follow-up.**

Seven publications on STN-DBS between 2010 and 2026, using the “old” generation DBS hardware, with 8-11 years (yrs) follow-up (F-U). Number of patients in each study; mean UPDRS-III scores off-med at baseline and mean UPDRS-III scores off-med on-stim at F-U; percentage motor improvement and percentage decrease in levodopa equivalent daily doses (LEDD).

| **Author (site)** | **Year** | **No**  **pat.** | **UPDRS-III**  **Off-med pre-op** | **UPDRS-III**  **Off-med On-stim** | **% change UPDRS III scores** | **% decrease**  **LEDD** | **F-U**  **years** |
| --- | --- | --- | --- | --- | --- | --- | --- |
| Aviles-Olmos^34^ (London) | 2014 | 12 | 57 | 36.2 | 36.4 | 48.6 | 8 |
| Zibetti^40^ (Torino) | 2011 | 14 | 51.3 | 29.5 | 42 | 39.3 | ≥9 |
| Chu^41^ (Guangzhou, China) | 2025 | 13 | 47.7 | 36.9 | 22.5 | 29.1 | ≥10 |
| Rizzone^42^ (Torino) | 2014 | 26 | 56.7 | 36.4 | 35.8 | 32.2 | 11 |
| Fasano^43^ (Toronto) | 2010 | 20 | 59.5 | 36.3 | 39 | 60.3 | 8 |
| Castrioto^44^ (Toronto) | 2011 | 18 | 50.2 | 38.8 | 22.7 | 36.3 | 10 |
| Ostrem^45^ (US multicenter) | 2026 | 19 | 43.2 | 28.8 | 33.3 | 39.3 | 10 |
| **Total no. of patients** | - | **122** |  |  |  |  |  |
| **Mean values ±SD**  **(range)** | - | - | **52.2±5.38**  **(43.2-59.5** | **34.7±3.60**  **(28.3-38.8)** | **33.1±7.09**  **(22.5-42)** | **40.7±9.83**  **(29.1-60.3)** | **(8-11)** |

**Supplemental Table 5:** Summary of data from STN-DBS studies using the “old” DBS hardware. Mean UPDRS-III scores and percentage changes in UPDRS-III and LEDD at various follow-up intervals. The no. of patients for each time period is the sum of patients of publication from Tables 1, 2, 3 and 4. Evidently, there is a great overlap of patients between publications from same center since same patients had been followed at various follow-up intervals, with an attrition in the number of patients the longer the follow-up.

| **Follow-up** | **No. pat** | **UPDRS-III pre-op**  **Off-med** | **UPDRS-III**  **post-op**  **Off-med On-stim** | **% UPDRS-III change** | **% LEDD decrease** |
| --- | --- | --- | --- | --- | --- |
| 6-12 months | 803 | 51.23±7.09 | 24.11±6.23 | 52.99±11.89 | 53.75±16.22 |
| 16 months-3 years | 482 | 47.16±10.05 | 28.38±6.26 | 39.93±11.01 | 50.6±17.77 |
| 4-6 years | 381 | 50.7±8.54 | 26.4±4.74 | 46.9±10.61 | 47.5±11.06 |
| 8-11 years | 122 | 52.2±5.38 | 34.7±3.60 | 33.1±7.09 | 40.7±9.83 |

**Supplemental Table 6: “new” DBS; 3-12 month follow-up.**

Nine publications on STN-DBS between 2012 and January 2026, using “modern” DBS hardware, with a 3-12 months follow-up (F-U). Number of patients in each study; mean±SD UPDRS-III scores off-med at baseline and mean±SD UPDRS-III scores off-med on-stim at follow-up; percentage motor improvement and percentage decrease in levodopa equivalent daily doses (LEDD).

| **Author (site)** | **Year** | **No**  **pat.** | **UPDRS-III**  **Off-med pre-op** | **UPDRS-III**  **Off-med On-stim** | **% change UPDRS-III scores** | **% decrease**  **LEDD** | **F-U**  **months** | **Comments** |
| --- | --- | --- | --- | --- | --- | --- | --- | --- |
| Kübler^46^ (Berlin) | 2023 | 203 | 45.3 ±17.55 | 28.3 ±16.91 | 35.8 | --- | 12 | Mixed system |
| Timmermann^47^ (Germany MC) | 2015 | 39 | 37.4 ±$8.9$ | 13.7 ±6.9 | 63.3 | 64 | 12 | BSc |
| Okun^48^ (US MC) | 2012 | 101 | 40.8 ±10.8 | 24.8 ±10.1 | 39 | 34 | 3 | St-Jude |
| Vitek^49^ (US MC) | 2020 | 118 | 36.9 ±10.83 | 25.1 ±10.99 | 32 | --- | 3 | BSc double-blind |
| Gharabaghi^50^ (Tübingen) | 2026 | 41 | 43.3 ±16.9 | 25.1 ±17.1 | 42 | 13 | 12 | Abbott in person progr. |
| Gharabaghi^50^(Tübingen) | 2026 | 38 | 46.8 ±15.8 | 29.1 ±12 | 37.8 | 7.4 | 12 | Abbott remote progr. |
| Schnitzler^51^ (international MC) | 2022 | 234 | 36.3 ±12.9 | 22.4 ±10.4 | 38 | 43 | 3-6 | Abbott direct. & omni. |
| Kallel^52^ (Grenoble) | 2025 | 33 | 43.1±**13.34** | 26.4 ±**12.58** | 38.7 | 53.2 | 12 | BSc omni. progr. |
| Kallel^52^ (Grenoble) | 2025 | 27 | 49.6 ±**13.34** | 28.6 ±**12.58** | 42.3 | 48.3 | 12 | BSc direct. progr. |
| Gharabaghi^53^ (Tübingen) | 2024 | 19 | 39.6 ±12.9 | 28.6 ±12.9 | 27.7 | --- | 6 | BSc omni. progr. |
| Gharabaghi^53^ (Tübingen) | 2024 | 19 | 39.6 ±12.9 | 32.4 ±15.7 | 18.2 | --- | 6 | BSc direct. progr. |
| Aldred^54^ (US MC) | 2025 | 46 | 48.5 ±14 | 24.4 ±12.8 | 46.4 | --- | 12 | XT-based progr. |
| **Total no. of patients** |  | **918** |  |  |  |  |  |  |
| **Mean values±SD**  **(range)** |  |  | **42.26±4.36**  **(36.3-49.6)** | **25.74±4.45**  **(13.7-32.4)** | **38.43±10.34**  **(18.2-63.3)** | **37.55±19.32**  **(7.4-64)** | **(3-12)** |  |

**Abbreviations:** MC: Multicentre; BSc: Boston Scientific; progr: programming. Direct: directional programming; omni: omnidirectional programming; XT: Imaging platform from BSc and BrainLab.

**REFERENCES for Tables:**

1. Krack P, Pollak P, Limousin P, Hoffmann D, Xie J, Benazzouz A, et al.: Subthalamic nucleus or internal pallidal stimulation in young onset Parkinson’s disease. Brain 1998; 121: 451-457.

2. Limousin P, Krack P, Pollak P, Benazzouz A, Ardouin C, Hoffmann D, et al. Electrical stimulation of the subthalamic nucleus in advanced Parkinson’s disease. N Engl J Med 1998; 339: 1105-1111.

3. Kumar R, Lozano AM, Kim YJ, Hutchison WD, Sime E, Halket E, et al. Double-blind evaluation of subthalamic nucleus deep brain stimulation in advanced Parkinson’s disease. Neurology 1998; 51: 850-585.

4. Moro E, Scerrati M, Romito LM, Roselli R, Tonali P, Albanese A. Chronic subthalamic nucleus stimulation reduces medication requirements in Parkinson’s disease. Neurology 1999; 53: 85-90.

5. Pinter MM, Alesch F, Murg M, Seiwald M, Helscher RJ, Binder H. Deep brain stimulation of the subthalamic nucleus for control of extrapyramidal features in advanced idiopathic Parkinson’s disease: one year follow-up. J Neural Transm 1999; 106: 693-709.

6. Romito LM, Scerrati M, Contarino MF, Bentivoglio AR, Tonali P, Albanese A. Long-term follow up of subthalamic nucleus stimulation in Parkinson's disease. Neurology 2002; 58(10): 1546-1550.

7. Anderson VC, Burchiel KJ, Hogarth P, Favre J, Hammerstad JP. Pallidal vs subthalamic nucleus deep brain stimulation in Parkinson disease. Arch Neurol. 2005; 62(4): 554-560.

8. Houeto JL, Damier P, Bejjani PB, Staedler C, Bonnet AM, Arnulf I, et al. Subthalamic stimulation in Parkinson disease: a multidisciplinary approach. Arch Neurol 2000; 57: 461-465.

9. Fraix V, Pollak P, Van Blercom N, Xie J, Krack P, Koudsie A, et al. Effect of subthalamic nucleus stimulation on levodopa-induced dyskinesia in Parkinson’s disease. Neurology 2000; 55: 1921-1923.

10. Molinuevo JL, Valldeoriola F, Tolosa E, Rumia J, Valls-Sole J, Roldan II, et al. Levodopa withdrawal after bilateral subthalamic nucleus stimulation in advanced Parkinson disease. Arch Neurol 2000; 57: 983-988.

11. Volkmann J, Allert N, Voges J, Weiss PH, Freund HJ, Sturm V. Safety and efficacy of pallidal or subthalamic nucleus stimulation in advanced PD. Neurology 2001; 56: 548-551.

12. The Deep-Brain Stimulation For Parkinson’s Disease Study Group. Deep-brain stimulation of the subthalamic nucleus or the pars interna of the globus pallidus in Parkinson’s disease. N Engl J Med 2001; 345: 956–963.

13. Rodriguez-Oroz MC, A Gorospe, J Guridi, E Ramos, G Linazasoro, M Rodriguez-Palmero, et al. Bilateral deep brain stimulation of the subthalamic nucleus in Parkinson's disease. Neurology 2000; 55(12 Suppl 6): S45-51.

14. Ostergaard K, Sunde N, Dupont E: Effects of bilateral stimulation of the subthalamic nucleus in patients with severe Parkinson’s disease and motor fluctuations. Mov Disord 2002; 17: 693-700.

15. Simuni T, Jaggi JL, Mulholland H, Hurtig HI, Colcher A, Siderowf AD, et al: Bilateral stimulation of the subthalamic nucleus in patients with Parkinson disease: a study of efficacy and safety. J Neurosurg 2002; 96: 666-672.

16. Bejjani BP, Dormont D, Pidoux B, Yelnik J, Damier P, Arnulf I, et al. Bilateral subthalamic stimulation for Parkinson's disease by using three-dimensional stereotactic magnetic resonance imaging and electrophysiological guidance. J Neurosurg 2000; 92(4): 615-625.

17. Deuschl G, Schade-Brittinger C, Krack P, Volkmann J, Schäfer H, Bötzel K, et al. A randomized trial of deep-brain stimulation for Parkinson's disease. N Engl J Med 2006; 355(9): 896-908.

18. Williams A, Gill S, Varma T, Jenkinson C, Quinn N, Mitchell R, et al. Deep brain stimulation plus best medical therapy versus best medical therapy alone for advanced Parkinson's disease (PD SURG trial): a randomised, open-label trial. Lancet Neurol 2010; 9(6) :581-591.

19. De Gaspari D, Siri C, Landi A, Cilia R, Bonetti A, Natuzzi F, et al. Clinical and neuropsychological follow up at 12 months in patients with complicated Parkinson's disease treated with subcutaneous apomorphine infusion or deep brain stimulation of the subthalamic nucleus. J Neurol Neurosurg Psychiatry 2006; 77(4): 450-453.

20. Odekerken VJ, van Laar T, Staal MJ, Mosch A, Hoffmann CF, Nijssen PC, et al. Subthalamic nucleus versus globus pallidus bilateral deep brain stimulation for advanced Parkinson’s disease (NSTAPS study): a randomised controlled trial. Lancet Neurol 2013; 12(1): 37-44.

21. Follett KA, Weaver FM, Stern M, Hur K, Harris CL, Luo P, Marks WJ Jr, Rothlind J, Sagher O, Moy C, Pahwa R, Burchiel K, Hogarth P, Lai EC, Duda JE, Holloway K, Samii A, Horn S, Bronstein JM, Stoner G, Starr PA, Simpson R, Baltuch G, De Salles A, Huang GD, Reda DJ; CSP 468 Study Group. Pallidal versus subthalamic deep-brain stimulation for Parkinson's disease. N Engl J Med. 2010 Jun 3;362(22):2077-91.

22. Wider C, Pollo C, Bloch J, Burkhard PR, Vingerhoets FJ. Long-term outcome of 50 consecutive Parkinson's disease patients treated with subthalamic deep brain stimulation. Parkinsonism Relat Disord. 2008;14(2):114-9.

23. Kleiner-Fisman G, Fisman DN, Sime E, Saint-Cyr JA, Lozano AM, Lang AE. J Long-term follow up of bilateral deep brain stimulation of the subthalamic nucleus in patients with advanced Parkinson disease. Neurosurg 2003; 99(3): 489-495.

24. Vingerhoets FJ, Villemure JG, Temperli P, Pollo C, Pralong E, Ghika J. Subthalamic DBS replaces levodopa in Parkinson's disease: two-year follow-up. Neurology 2002; 58(3): 396-401.

25. Pahwa R, Wilkinson SB, Overman J, Lyons KE. Bilateral subthalamic stimulation in patients with Parkinson disease: long-term follow up. J Neurosurg 2003; 99(1): 71-77.

26. Weaver FM, Follett KA, Stern M, Luo P, Harris CL, Hur K, et al. Randomized trial of deep brain stimulation for Parkinson disease: thirty-six-month outcomes. Neurology 2012; 79(1): 55-65.

27. Herzog J, Volkmann J, Krack P, Kopper F, Pötter M, Lorenz D, et al. Two-year follow-up of subthalamic deep brain stimulation in Parkinson's disease. Mov Disord 2003;18(11): 1332-1337.

28. Odekerken VJ, Boel JA, Schmand BA, de Haan RJ, Figee M, van den Munckhof P, et al. GPi vs STN deep brain stimulation for Parkinson disease: Three-year follow-up. Neurology 2016; 86(8): 755-761.

29. Schuepbach WM, Rau J, Knudsen K, Volkmann J, Krack P, Timmermann L, et al. Neurostimulation for Parkinson's disease with early motor complications. N Engl J Med 2013;368(7): 610-622.

30. Rodriguez-Oroz MC, Zamarbide I, Guridi J, Palmero MR, Obeso JA. Efficacy of deep brain stimulation of the subthalamic nucleus in Parkinson's disease 4 years after surgery: double blind and open label evaluation. J Neurol Neurosurg Psychiatry 2004; 75(10):1382-1385.

31. Rodriguez-Oroz MC, Obeso JA, Lang AE, Houeto JL, Pollak P, Rehncrona S, et al. Bilateral deep brain stimulation in Parkinson's disease: a multicentre study with 4 years follow-up. Brain 2005; 128(Pt 10): 2240-2249.

32. Moro E, Lozano AM, Pollak P, Agid Y, Rehncrona S, Volkmann J, et al. Long-term results of a multicenter study on subthalamic and pallidal stimulation in Parkinson's disease. Mov Disord 2010; 25(5): 578-586.

33. Krack P, Batir A, Van Blercom N, Chabardes S, Fraix V, Ardouin C, et al. Five-year follow-up of bilateral stimulation of the subthalamic nucleus in advanced Parkinson's disease. N Engl J Med 2003;349(20): 1925-1934.

34. Aviles-Olmos I, Kefalopoulou Z, Tripoliti E, Candelario J, Akram H, Martinez-Torres I, et al. Long-term outcome of subthalamic nucleus deep brain stimulation for Parkinson's disease using an MRI-guided and MRI-verified approach. J Neurol Neurosurg Psychiatry 2014; 85(12): 1419-1425.

35. Gervais-Bernard H, Xie-Brustolin J, Mertens P, Polo G, Klinger H, Adamec D, et al. Bilateral subthalamic nucleus stimulation in advanced Parkinson’s disease: five year follow-up. J Neurol 2009; 256(2): 225-233.

36. Jiang LL, Liu JL, Fu XL, Xian WB, Gu J, Liu YM, et al. Long-term Efficacy of Subthalamic Nucleus Deep Brain Stimulation in Parkinson's Disease: A 5-year Follow-up Study in China. Chin Med J 2015; 128: 2433-2438.

37. Li J, Zhang Y, Li Y. Long-term follow-up of bilateral subthalamic nucleus stimulation in Chinese Parkinson's disease patients. Br J Neurosurg 2015; 29: 329-333.

38. Schüpbach WMM, Chastan N, Welter ML, Houeto JL, Mesnage V, Bonnet AM, et al. Stimulation of the subthalamic nucleus in Parkinson’s disease: a 5 year follow up. J Neurol Neurosurg Psychiatry 2005; 76: 1640-1644.

39. Kishore A, Rao R, Krishnan S, Panikar D, Sarma G, Sivasanakaran MP, et al. Long-term stability of effects of subthalamic stimulation in Parkinson’s disease: Indian Experience. Mov Disord 2010; 25: 2438-2444.

40. Zibetti M, Merola A, Rizzi L, Ricchi V, Angrisano S, Azzaro C, et al. Beyond nine years of continuous subthalamic nucleus deep brain stimulation in Parkinson's disease. Mov Disord 2011; 26(13): 2327-2334.

41. Chu J, Wu L, Jiang L, Chen J, Gu J, Qian H, et al. Long-term efficacy of deep brain stimulation in Parkinson's disease: over 10-year follow-up and insights into the "DBS honeymoon". Ther Adv Neurol Disord 2025; 18: 17562864251388840.

42. Rizzone MG, Fasano A, Daniele A, Zibetti M, Merola A, Rizzi L, et al. Long-term outcome of subthalamic nucleus DBS in Parkinson's disease: from the advanced phase towards the late stage of the disease? Parkinsonism Relat Disord 2014; 20(4) :376-381.

43. Fasano A, Romito LM, Daniele A, Piano C, Zinno M, Bentivoglio AR, et al. Motor and cognitive outcome in patients with Parkinson’s disease 8 years after subthalamic implants. Brain 2010; 133(9): 2664-2676.

44. Castrioto A, Lozano AM, Poon YY, Lang AE, Fallis M, Moro E. Ten-year outcome of subthalamic stimulation in Parkinson disease: a blinded evaluation. Arch Neurol 2011; 68(12) :1550-1556.

45. Ostrem JL, Luo P, Weaver FM, Follett K, Rothlind J, Galifianakis NB, et al. 10-year clinical outcomes of subthalamic nucleus versus pallidal deep brain stimulation for Parkinson's disease: VA/NINDS CSP #468F. Front Neurol. 2026; 16: 1728999.

46. Kübler D, Astalosch M, Gaus V, Krause P, de Almeida Marcelino AL, Schneider GH, et al. Gender-specific outcomes of deep brain stimulation for Parkinson's disease - results from a single movement disorder center. Neurol Sci 2023; 44(5): 1625-1631.

47. Timmermann L, Jain R, Chen L, Maarouf M, Barbe MT, Allert N, et al. Multiple-source current steering in subthalamic nucleus deep brain stimulation for Parkinson's disease (the VANTAGE study): a non-randomised, prospective, multicentre, open-label study. Lancet Neurol 2015; 14(7): 693-701.

48. Okun MS, Gallo BV, Mandybur G, Jagid J, Foote KD, Revilla FJ, et al. Subthalamic deep brain stimulation with a constant-current device in Parkinson’s disease: an open-label randomised controlled trial. Lancet Neurol 2012; 11(2): 140-149.

49. Vitek JL, Jain R, Chen L, Tröster AI, Schrock LE, House PA, et al. Subthalamic nucleus deep brain stimulation with a multiple independent constant current-controlled device in Parkinson's disease (INTREPID): a multicentre, double-blind, randomised, sham-controlled study. Lancet Neurol 2020;19(6): 491-501.

50. Gharabaghi A, Groppa S, Casas E, Schnitzler A, Muñoz-Delgado L, Marshall VL, et al. Real-world multicenter assessment of sustained clinical outcomes after digital deep brain stimulation. NPJ Digit Med 2026; 9(1): 133.

51. Schnitzler A, Mir P, Brodsky MA, Verhagen L, Groppa S, Alvarez R, et al. Directional deep brain stimulation for Parkinson’s disease: results of an international crossover study with randomized, double-blind primary endpoint. Neuromodulation 2022; 25(6): 817-828.

52. Kallel M, De Schlichting E, Fraix V, Castrioto A, Moro E, Cordier L, et al. Comparing Directional and Omnidirectional Deep Brain Stimulation in Parkinson's Disease Patients. Stereotact Funct Neurosurg 2025; 103(2): 111-123.

53. Gharabaghi A, Cebi I, Leavitt D, Scherer M, Bookjans P, Brunnett B, et al. Randomized crossover trial on motor and non-motor outcome of directional deep brain stimulation in Parkinson's disease. NPJ Parkinsons Dis. 2024; 10(1): 204.

54. Aldred JL, Zesiewicz T, Okun MS, Ramirez-Zamora A, Vaou OE, Verhagen Metman L, et al. Sustained Therapeutic Benefits Using Image-Guided Programming at Activation of Deep Brain Stimulation for Parkinson's Disease. Mov Disord Clin Pract 2025;12(11): 1821-1830.

55. Starr PA, Shivacharan RS, Goldberg E, Tröster AI, House PA, Giroux ML, et al. Five-Year Outcomes from Deep Brain Stimulation of the Subthalamic Nucleus for Parkinson Disease. JAMA Neurol 2025; 82(11): 1181-1190.
