# Supplementary material for: Hardware Sophistications in Subthalamic Nucleus Deep Brain Stimulation for Parkinson's Disease; Is the Juice Worth the Squeeze?
Source: Mov Disord Clin Pract. 2026 Jul 2:10.1002/mdc3.70725. Online ahead of print. doi: 10.1002/mdc3.70725 (PMC13337137; doi:10.1002/mdc3.70725)
Supplement: Supplementary file 2 — Supplemental File S2. Meta‐analysis. Figure S1. Preoperative baseline off‐med Unified Parkinson's Disease Rating Scale Part III (UPDRS III) scores in old deep brain stimulation (DBS) patients (blue) and preoperative baseline off‐med UPDRS III scores in new DBS patients (green). Figure S2. Postoperative off‐med Unified Parkinson's Disease Rating Scale Part III (UPDRS III) scores in old deep brain stimulation (DBS) patients (blue) and postoperative off‐med on‐stim UPDRS III scores in new DBS patients (green). Table S1. The meta‐analytic moderator analysis (old deep brain stimulation [DBS] vs. new DBS at baseline). Estimate is based on a random effect (RE) model using the DerSimonian–Laird approach. CI, confidence interval. Table S2. The meta‐analytic moderator analysis (old deep brain stimulation [DBS] vs. new DBS at follow‐up). Estimate is based on a random effect (RE) model using the DerSimonian–Laird approach. CI, confidence interval. [file MDC3-9999-0-s003.docx]

**SUPPLEMENTAL FILE 2: Meta-analysis.**

We have performed a subgroup meta-analysis using a random-effects model (DerSimonian-Laird approach) to account for between study heterogeneity. For this, we used the statistical tool “Jamovi”, version 2.7.24.0, to compare preoperative baseline off-med UPDRS III scores in old DBS patients with preoperative baseline off-med UPDRS III scores in new DBS patients. Postoperatively we compared on-stim off-med UPDRS III scores in old DBS patients with on-stim off-med UPDRS III scores in new DBS patients, the moderator in both comparisons being thus old DBS versus new DBS.

We obtained two sets of forest plots and effect sizes, as shown below in Supplemental Figure 1 and Supplemental Table 1.


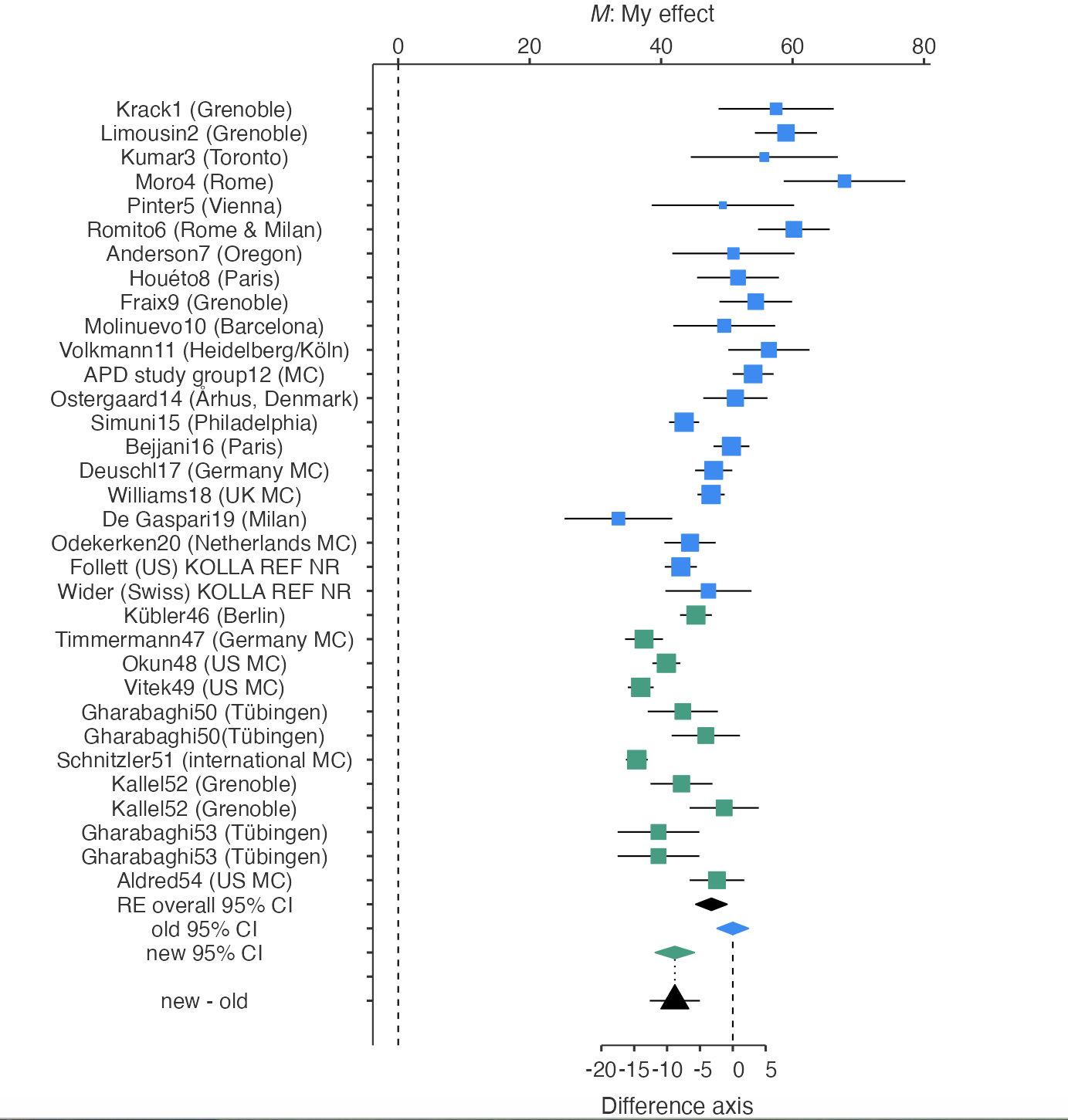


**Supplemental Figure 1:** Preoperative baseline off-med UPDRS III scores in old DBS patients (BLUE) and preoperative baseline off-med UPDRS III scores in new DBS patients (GREEN)

**Supplemental Table 1** shows the meta-analytic moderator analysis (old DBS vs new DBS at baseline). Estimate is based on a random effect (RE) model using the DerSimonian-Laird approach. C.I.= Confidence Interval.

| Level | Mean | 95% C.I. Lower Limit | 95% C.I.  Upper Limit |
| --- | --- | --- | --- |
| New DBS | 42.09 | 39.1 | 45.07 |
| Old DBS | 50.91 | 48.5 | 53.29 |
| new – old | –8.82 | –12.6 | -5.01 |

The table above shows a 95% CI between -12.6 and -5.01, meaning a highly significant difference in preoperative baseline characteristics between the two cohorts: Patients undergoing the old DBS procedure presented with significantly higher UPDRS III off-med scores than patients who had the new DBS.

A similar meta-analytic statistic procedure of postoperative off-med on-stim UPDRS III scores in old DBS patients (BLUE) versus postoperative off-med on-stim UPDRS III scores in new DBS patients (GREEN), is shown below in Supplemental Figure 2 and Supplemental Table 2.


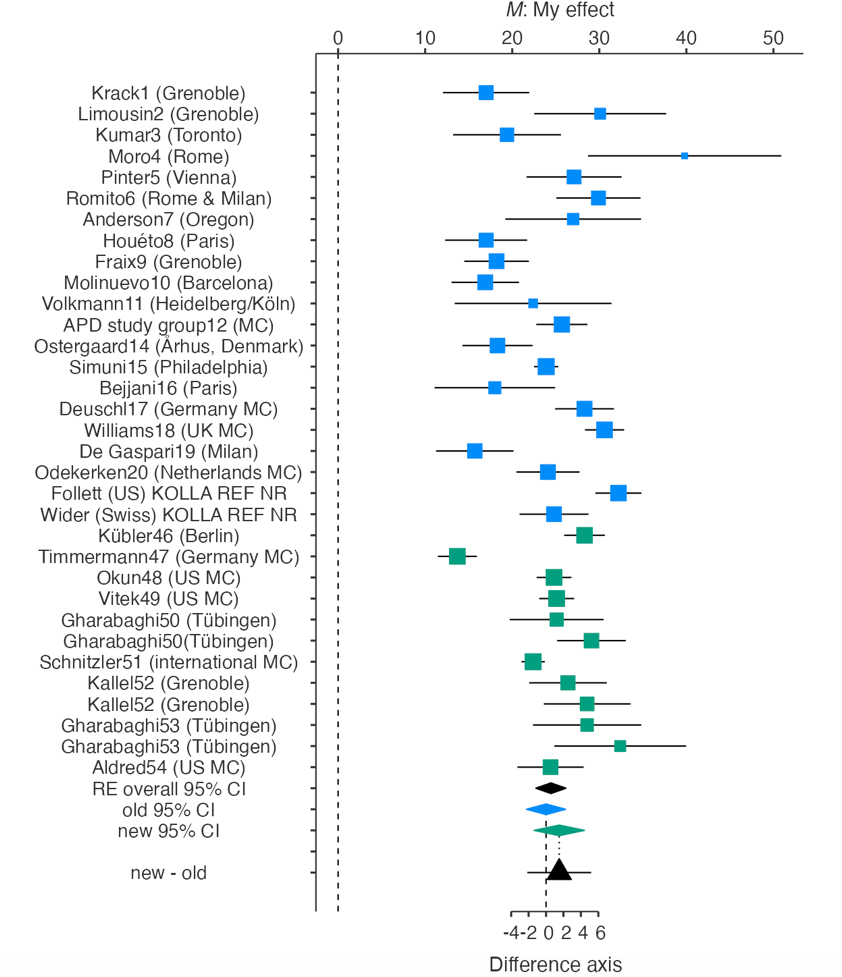


**Supplemental Figure 2:** Postoperative off-med UPDRS III scores in old DBS patients (BLUE) and postoperative off-med on-stim UPDRS III scores in new DBS patients (GREEN)

**Supplemental Table 2:** shows the meta-analytic moderator analysis (old DBS vs new DBS at follow-up). Estimate is based on a random effect (RE) model using the DerSimonian-Laird approach. C.I.= Confidence Interval.

| Level | Mean | 95% C.I. Lower Limit | 95% C.I.  Upper Limit |
| --- | --- | --- | --- |
| New DBS | 25.40 | 22.50 | 28.29 |
| Old DBS | 23.88 | 21.63 | 26.13 |
| new – old | 1.52 | –2.15 | 5.19 |

Postoperatively, there was no significant difference between UPDRS III scores in patients who had old DBS compared to those who had new DBS.

In summary, for the comparison of preoperative UPDRS III at baseline between old DBS patients and new DBS patients, the moderator analysis revealed a statistically significant difference between the two groups (Mdiff = -8.82; 95% CI [-12.60,- 5.01]; p<0.001). Patients in the studies evaluating the new DBS presented with significantly lower UPDRS scores (M = 42.09; 95% CI [39.10, 45.07]) compared to those in the old DBS group (M = 50.91; 95% CI [48.50, 53.29]). This indicates that the patients receiving the new DBS had less severe symptoms prior to surgery.

For the comparison of postoperative UPDRS III, the moderator analysis showed no statistically significant difference in UPDRS scores between the old DBS and New DBS (Mdiff = 1.52; 95% CI [- 2.15, 5.19]; p = 0.37). Patients with new DBS had a mean score of 25.40 (95% CI [22.50, 28.29]), while patients with old DBS had a mean score of 23.88 (95% CI [21.63, 26.13]). Thus, despite the initial difference at baseline, patients with old DBS achieved a greater absolute reduction in symptom severity, leading to comparable UPDRS III outcomes between the two groups at follow-up.
